# Sex-Based Representation and Outcome Reporting in Thoracolumbar Burst Fracture Research: A PRISMA-ScR Scoping Review

**DOI:** 10.3390/jcm15197478

**Published:** 2026-09-25

**Authors:** Sonja Häckel, Anna Veenstra, Helena Mengolian, Yuliia Divnych, Julia D. Jenter, Marc von Gernler, Samuel F. Schaible, Fabienne Pohle, Tamara Babasiz, Nikolaus Kernich, Christoph E. Albers, Christian Tinner

**Affiliations:** 1 Department of Orthopaedic Surgery and Traumatology, Inselspital, University Hospital Bern, University Bern, Freiburgstrasse 18, 3010 Bern, Switzerland; 2 Graduate School of Health Sciences, University Bern, Mittelstrasse 43, 3012 Bern, Switzerland; 3 Department of Traumatology, University Hospital Zurich, University of Zurich, Rämistrasse 71, 8006 Zurich, Switzerland; 4 Orthopädische Klinik Luzern AG, Sankt-Anna-Strasse 28, 6006 Lucerne, Switzerland; 5 Medical Library, University Library of Bern, University of Bern, Baltzerstrasse 4, 3012 Bern, Switzerland; 6 Department for Orthopaedic and Trauma Surgery, University Hospital of Cologne, Kerpener Strasse. 62, 50937 Lindenthal, Germany; 7 Spine Unit, Sonnenhofspital, Buchserstrasse 30, 3006 Bern, Switzerland

**Keywords:** thoracolumbar burst fracture, spinal fractures, spinal injuries, sex factors, women, treatment outcome, scoping review, sex-disaggregated reporting, SAGER guidelines

## Abstract

**Background/Objectives:** Thoracolumbar burst fracture research has traditionally been male-skewed, but it remains unclear how often women are represented and whether outcomes are reported separately for women and men. This scoping review mapped sex-based representation and outcome reporting in clinical studies of adult traumatic thoracolumbar burst fractures. **Methods:** We conducted a prospectively planned scoping review in accordance with the PRISMA Extension for Scoping Reviews (PRISMA-ScR). MEDLINE, Embase, CINAHL, Global Health, CENTRAL, ClinicalTrials.gov, and ICTRP were searched initially on 8 October 2024 and updated on 8 January 2026. Randomized, prospective, and retrospective clinical reports of adult traumatic thoracolumbar burst fractures were eligible. Reports of pathologic or osteoporotic fractures, pediatric cohorts, nonoriginal reports, protocols without results, and reports with fewer than 11 participants were excluded. Data were charted descriptively, with particular attention to the number and proportion of women, sex-related reporting, and reports identifying differences between women and men. **Results:** Of 8285 records identified, 491 reports were included, representing an aggregate reported sample of 44,147 participants. Most reports were retrospective (296/491, 60.3%) and evaluated operative techniques (410/491, 83.5%). The number or proportion of women was available in 461 reports (93.9%). The median report-level proportion of women was 36.0% (interquartile range, 29.9–43.3%), and 300 of 461 reports (65.1%) included fewer than 40% women. Only 52 of 491 reports (10.6%) reported any sex-related result, and 10 of 491 reports (2.0%) reported a between-sex treatment-outcome difference. **Conclusions:** Women are routinely counted but rarely analyzed separately in thoracolumbar burst fracture reports. The available sex-related outcome data are sparse, heterogeneous, and primarily hypothesis-generating. Future studies should report outcomes separately for women and men.

## 1. Introduction

Thoracolumbar burst fractures are common traumatic spinal injuries, and there remains debate regarding surgical versus conservative treatment, fixation strategies and follow-up care. Extensive epidemiological studies and recent systematic reviews confirm that these injuries predominantly occur following high-energy trauma and that treatment approaches remain heterogeneous depending on the institution and region. In most clinical study series, men are affected more frequently than women (approx. 65% vs. 35%), which is likely due to greater exposure to risk factors such as road traffic accidents, workplace accidents and high-risk sports [1,2,3]. This epidemiological pattern is also reflected in clinical study populations. However, numerical representation alone does not indicate whether sex is considered in the analysis or interpretation of outcomes. Differences between women and men are increasingly recognised as clinically relevant determinants of clinical presentations, recovery and patient-reported outcomes following musculoskeletal injuries and spinal surgery. However, the evidence on sex-specific determinants remains limited and heterogeneous, particularly among trauma patients [4]. The recommendations of the ‘Sex and Gender Equity in Research’ (SAGER) initiative call for explicit consideration of biological sex and social gender in study design, analysis, results and interpretation [5].

In the case of thoracolumbar burst fractures in particular, isolated reports suggest that outcomes may differ between women and men. Maior et al. reported differences in terms of health-related quality of life and disability one year after surgery for thoracolumbar burst fractures, while a recent prospective multicentre study suggested possible differences in treatment response following surgical and non-surgical management [6,7]. These observations are clinically relevant but should not be interpreted as definitive treatment recommendations, as they are derived from a small, heterogeneous subset of the literature.

The aim of this scoping review was therefore to map sex-based representation and outcome reporting in the clinical literature on adult traumatic thoracolumbar burst fractures. Specifically, we sought to (1) describe the representation of women in the included reports, (2) determine how frequently outcomes were analysed or reported in relation to sex, and (3) characterise reports identifying differences in treatment outcomes between women and men. The review was designed to identify gaps in sex-disaggregated reporting and analysis rather than to estimate pooled treatment effects or derive sex-specific treatment recommendations.

## 2. Materials and Methods

### 2.1. Design and Reporting Framework

This review was conducted as a scoping review and reported in accordance with the PRISMA Extension for Scoping Reviews (PRISMA-ScR) [8]. The review methods and eligibility criteria were defined a priori. The database-specific search strategies were subsequently made publicly available on searchRxiv [9,10,11,12,13,14,15].

### 2.2. Inclusion Criteria

Reports were eligible if they included adults with traumatic thoracolumbar burst fractures treated either surgically or conservatively and reported primary clinical data. Where the AO Spine classification was specified, the target morphology was a type A3 or A4 thoracolumbar burst fracture. Reports limited to type A0–A2, A1, B or C injuries were excluded. Mixed fracture cohorts were eligible when more than 50% of the analysed cohort consisted of A3/A4 thoracolumbar burst fractures, or when data for the eligible A3/A4 subgroup were separately extractable. Otherwise, the report was excluded. The same principle was applied to older fracture classification systems, including equivalent Magerl burst-fracture categories. Randomised controlled trials and prospective or retrospective clinical reports with at least 11 patients were eligible. Reports with fewer than 11 patients were excluded. Reports of pathological or osteoporotic fractures, paediatric populations, non-original reports, conference abstracts, protocols or trial registrations without results, and reports in which traumatic thoracolumbar burst fractures could not be distinguished from other fracture patterns were excluded.

### 2.3. Information Sources and Search Strategy

A systematic literature search was conducted in MEDLINE (Ovid), Embase (Ovid), CENTRAL (Cochrane Library), CINAHL (EBSCO), Global Health (Ovid), ClinicalTrials.gov and the WHO International Clinical Trials Registry Platform. An initial MEDLINE strategy was developed by a medical information specialist and tested against a set of core references. Database-specific controlled vocabularies and free-text terms were then adapted for each source. No restrictions regarding language, publication year, or study design were applied at the database-search stage. The search combined terms related to thoracolumbar fractures, surgical and conservative treatment, and outcomes. Reports conducted exclusively in animals were excluded from MEDLINE and Embase using double-negative filters for human reports. The initial search was conducted on 8 October 2024. All searches were updated on 8 January 2026, with the exception of ‘Global Health’, which was no longer available at that time. Detailed search strategies are provided in the Supplementary Materials and are publicly available on searchRxiv [9,10,11,12,13,14,15].

### 2.4. Screening and Data Extraction

All identified records were imported into Covidence (Covidence systematic review software. Veritas Health Innovation: Melbourne, Australia) for screening and data extraction. Duplicate records were automatically removed by Covidence; deduplication of the updated data was carried out using Deduklick [16]. Titles and abstracts were screened independently by two reviewers per record according to predefined eligibility criteria. Full-text assessment was likewise performed independently by two reviewers per report. Disagreements at either screening stage were resolved by discussion and consensus between the two reviewers. Inter-reviewer agreement statistics, including Cohen’s kappa, were generated by Covidence and exported for descriptive reporting. Reasons for exclusion were documented during the full-text review.

Data were extracted using a structured Covidence extraction form that was piloted before full data extraction. Data extraction was performed by one reviewer and subsequently checked by a second reviewer. The extracted variables included publication year, study location, study design, treatment category, total sample size, number and proportion of women, whether sex-related results were reported, whether treatment outcomes differed between women and men, and any available sex-disaggregated data on age or intervention outcomes. No language restriction was applied during the database search; however, reports that could not be reliably assessed at full-text stage because an accessible translation was unavailable were excluded and recorded as language-related exclusions.

### 2.5. Terminology: Biological Sex, Social Gender, Women and Men

Because the primary reports rarely specified how participant sex or gender was ascertained, construct-level interpretation was limited. In this review, we use “sex-related” as the primary term to describe analyses based on the binary female/male or women/men categories reported by the original reports and extraction form. We do not infer gender identity or broader sociocultural gender constructs unless these were explicitly assessed by the source study. The term “gender” is therefore reserved for discussion of broader reporting frameworks, including the SAGER recommendations, and for cases in which the original source used that terminology.

### 2.6. Methodological Context and Level of Evidence

The primary aim of this scoping review was to map representation and reporting practices, rather than to determine the pooled efficacy of a treatment. Accordingly, a formal risk-of-bias assessment using an intervention-specific tool was not undertaken. For reports contributing sex-related findings, methodological context was recorded descriptively, including study design, treatment approach, sample size, whether the sex-related analysis was prespecified or secondary, and, where available, effect estimates, measures of uncertainty, and adjustment for potential confounders. These findings were interpreted as hypothesis-generating rather than as evidence supporting sex-specific treatment recommendations.

### 2.7. Data Synthesis

The data were described using descriptive analysis. Obvious variations in free-text responses regarding study design and in ‘yes/no’ fields were standardised prior to analysis. Where both the number of women and the total sample size were available, the proportion of women at report level was derived from these figures; reported percentages were used only when the number of women was unavailable. Apparent inconsistencies between participant counts and reported percentages identified during the final data audit were checked against the source publications and corrected in the analysis dataset. The two sex-related variables, whether any sex-related result was reported and whether a difference in treatment outcome between women and men was reported, were analysed separately. The representation of women was summarised as the median proportion of women at report level and also grouped as <40%, 40–49.9%, and ≥50% women. The aggregate proportion of women was calculated from the summed numbers of women and participants in reports with internally consistent data. Publication years were categorised into the groups ≤1999, 2000–2009, 2010–2019 and 2020–2025 in order to examine temporal patterns. Descriptive analyses were performed in R using RStudio (version 2023.06.1+524; Posit Software, PBC, Boston, MA, USA). As the available sex-disaggregated results were sparse and heterogeneous, no meta-analysis was conducted. Generative AI-assisted tools were used during manuscript revision for numerical consistency checking and preparation of tables and figures; all outputs were verified by the authors against the final extraction dataset and source publications.

## 3. Results

### 3.1. Study Selection

The search identified 8285 records from databases and trial registries. After removal of 2552 duplicates, including 2530 duplicates identified in Covidence and 22 removed manually, 5733 records underwent title and abstract screening. Across all reviewer pairs, agreement at this stage was 83.9%, and the Covidence-generated Cohen’s kappa was 0.61. After exclusion of 4009 records at title and abstract screening, 1724 reports were sought for retrieval. Of these, 187 could not be retrieved, leaving 1537 reports for full-text eligibility assessment. A total of 1046 reports were excluded after full-text assessment, and 491 reports were included in the final synthesis (Figure 1).

### 3.2. Characteristics of the Included Reports

The included reports represented an aggregate reported sample of 44,147 participants and spanned the period from 1979 to 2025. Because reports rather than unique patient cohorts were the unit of analysis, this aggregate participant count may include participants described in companion publications. In total, 189 out of 491 reports (38.5%) were published between 2020 and 2025. Most reports were retrospective, followed by prospective non-randomized studies and randomized controlled trials. Reports on operative techniques were most common, whereas 42 reports (8.6%) compared operative and conservative treatment and 33 reports (6.7%) evaluated conservative treatment exclusively. Geographically, the evidence base was concentrated in Asia and Europe, with a smaller contribution from North America and other regions (Table 1).

### 3.3. Representation of Women

Participant composition was frequently reported. The number or proportion of women was available in 461 of 491 reports (93.9%). The median report-level proportion of women was 36.0% (interquartile range, 29.9–43.3%). Across these 461 reports, women accounted for 12,543 of 33,906 participants (37.0%). Overall, 300 of 461 reports (65.1%) included fewer than 40% women, 102 of 461 (22.1%) included 40–49.9% women, and 59 of 461 (12.8%) included at least 50% women (Table 2).

### 3.4. Frequency of Sex-Related Reporting

In contrast to the generally high reporting of participant composition, sex-related analyses were uncommon. Only 52 out of 491 reports (10.6%) reported any sex-related result. The proportion was highest among reports published between 2020 and 2025, with 28 out of 189 reports (14.8%), compared with 4 out of 43 (9.3%) published up to 1999, 6 out of 83 (7.2%) in 2000–2009, and 14 out of 176 (8.0%) in 2010–2019 (Figure 2).

### 3.5. Regional Patterns in Sex-Related Reporting

When considered by harmonized study region, Asia contributed the largest absolute number of reports with sex-related findings (29 reports), reflecting its larger overall literature volume. Europe showed the highest relative proportion of reports with a between-sex treatment-outcome difference, with such differences reported in 3.5% of European reports, compared with 1.3% of Asian reports, 0% of North American reports, and 3.6% of reports from other/multiregional/unclear regions (Table 3).

### 3.6. Between-Sex Outcome Differences

Among the 491 included reports, 10 (2.0%) were coded as reporting between-sex outcome differences, 181 (36.9%) were coded as not reporting such a difference, and in 300 (61.1%) the outcome-difference status was unknown or not reported. Within the subgroup of 52 reports that reported any sex-related result, 39 (75.0%) were retrospective and 41 (78.8%) focused on operative treatment techniques. Most sex-related findings arose from secondary or exploratory analyses rather than dedicated comparisons between women and men. The reported findings were heterogeneous and included patient-reported disability and quality of life, radiographic correction and loss of correction, vertebral recollapse, and functional recovery. The direction of the associations was not consistent across outcomes or treatment settings. For example, Dandurand et al. found that surgically treated women achieved clinically important ODI improvement faster than men, whereas nonoperatively treated women had a lower probability of improvement than nonoperatively treated men [6]. Maior et al. reported worse disability and several SF-36 domains in women one year after surgery [7]. Other reports identified sex associations with radiographic outcomes, although these were frequently secondary predictors and were not consistently maintained after multivariable adjustment (Table 4).

## 4. Discussion

This scoping review demonstrates a clear disconnect between routine reporting of participant composition and meaningful sex-disaggregated outcome reporting in thoracolumbar burst fracture research. Most included reports provided the number or proportion of women, and the median report-level proportion of women was 36%. This finding broadly reflects the known epidemiology of thoracolumbar burst fractures, which are more frequent in men and are commonly associated with high-energy mechanisms [1,2,3]. The more relevant finding is therefore not the numerical representation of women itself, but the limited use of sex-related analyses: only 52 of 491 reports (10.6%) reported any sex-related result, and only 10 of 491 reports (2.0%) reported a between-sex treatment-outcome difference. The main contribution of this review is that it identifies how limited the actually reported sex-disaggregated evidence remains within a clinically important and extensively studied fracture category. Contemporary discussions on thoracolumbar burst fracture care often focus on operative versus nonoperative management, posterior instrumentation strategies, vertebral augmentation, radiographic correction, complications, and patient-reported recovery [2,3,17,18]. However, the available literature rarely examines whether these outcomes are comparable between women and men. This matters because evidence syntheses and clinical recommendations may implicitly assume similar treatment response and recovery trajectories across sex categories, although this assumption has seldom been tested directly.

The ten reports identifying between-sex treatment-outcome differences should be interpreted cautiously. These reports were clinically heterogeneous, often retrospective, and frequently reported sex as one secondary predictor among several variables rather than as a pre-specified subgroup or interaction analysis. Accordingly, the present review does not support sex-specific treatment recommendations for thoracolumbar burst fractures. Rather, it identifies a clinically relevant evidence gap. The available findings, including those reported by Dandurand et al. and Maior et al., are best interpreted as hypothesis-generating signals that require confirmation in adequately designed prospective cohorts, registries, or secondary analyses of completed trials [6,7].

Individual reports suggest that recovery patterns and treatment response may differ between women and men, but these findings remain unconfirmed. Potential contributors include age distribution, injury mechanism, baseline disability, comorbidity burden, vertebral bone quality, pain experience, access to rehabilitation, socioeconomic factors, and treatment selection. Some included reports also suggest that patient-reported outcomes may differ even when radiographic findings appear similar [6,7,17,18]. These mechanisms are not mutually exclusive, and the current literature does not allow them to be separated. Bone quality is one plausible factor, particularly in peri- and postmenopausal women and older patients, because osteoporosis and reduced bone mineral density are well-established determinants of fracture risk and may influence fixation strength or implant-related complications [25,26]. However, this review excluded reports focused on osteoporotic fractures, and the included traumatic cohorts rarely reported bone density or menopausal status. Bone quality should therefore be considered a potential explanatory factor for future research, not a conclusion supported by the current evidence.

The findings also underline the need to distinguish carefully between biological sex and social gender. Most primary reports used binary categories such as female/male or women/men but did not describe how these variables were ascertained. Therefore, the results of this review should be interpreted as reflecting reported sex categories rather than gender identity or broader sociocultural gender constructs. This distinction is important because the SAGER recommendations and related guidance emphasize that authors should report whether sex, gender, or both were considered, how these variables were defined, and how they were incorporated into study design, analysis, and interpretation [5,27]. Future thoracolumbar fracture studies should make this distinction explicit.

### Strengths and Limitations

This scoping review used a comprehensive search of bibliographic databases and trial registries, applied methods and eligibility criteria defined a priori, and was reported in accordance with PRISMA-ScR [8]. The database-specific search strategies were made publicly available on searchRxiv. It captured a large body of thoracolumbar burst fracture literature and extracted several variables directly relevant to sex-related reporting, rather than limiting the assessment to whether women were present in study cohorts. The review also included platform-generated agreement statistics, which provided transparency regarding the reliability of title and abstract screening.

Several limitations should be acknowledged. First, the primary aim was to map the literature and reporting practices, not to estimate pooled treatment effects. No meta-analysis was performed because sex-disaggregated outcome data were sparse and clinically heterogeneous. Second, the analysis depended on the reporting quality of the original reports and the completeness of the extraction database. Many reports stated the number of women and men but did not provide outcomes separately for these groups. Because reports rather than unique patient cohorts were the unit of analysis, some participants may have been represented in more than one companion publication; therefore, aggregate participant counts should not be interpreted as numbers of unique individuals. Third, most source reports did not distinguish biological sex from gender identity and did not describe how sex or gender variables were recorded, limiting interpretation at the construct level. Fourth, interrater reliability could only be quantified for the title and abstract screening stage because the available full-text agreement export did not contain analyzable records. Although no language restriction was applied at the search stage, 141 reports could not be included because the available full text could not be reliably assessed and no accessible translation was available. This may have introduced language- or geography-related selection bias. In mixed fracture cohorts, sex-related analyses were not always reported separately for the eligible A3/A4 subgroup, which limits fracture-specific interpretation. Finally, although this review contextualized the methodological profile of the included literature, the identified sex-related signals should be regarded as hypothesis-generating in the absence of a formal risk-of-bias assessment across all included reports. The methodological profile of the underlying literature further limits interpretation. Most included reports were retrospective, and the few reports with sex-related outcomes often considered sex as a secondary or exploratory variable rather than as a pre-specified subgroup or interaction analysis. Even when between-sex differences were reported, the available data were usually insufficient to determine whether sex independently influenced outcomes or whether the observed associations reflected confounding by age, injury severity, injury mechanism, comorbidity burden, bone quality, treatment selection, rehabilitation access, or socioeconomic factors.

## 5. Conclusions

The thoracolumbar burst fracture literature largely reflects the known epidemiology of these injuries, with men representing the majority of reported participants. However, although women are routinely included and usually counted in reported cohorts, outcomes are rarely reported separately for women and men. Explicit between-sex comparisons remain uncommon, and the available sex-related outcome data are sparse, heterogeneous, and mainly derived from secondary or exploratory analyses. The current evidence is therefore not sufficient to guide sex-specific treatment decisions. Rather, it highlights a clear reporting and evidence gap. Future thoracolumbar burst fracture studies should distinguish biological sex from gender constructs, report baseline characteristics and clinical outcomes separately for women and men, and pre-specify sex-related analyses where clinically and methodologically appropriate.

## Figures and Tables

**Figure 1:**
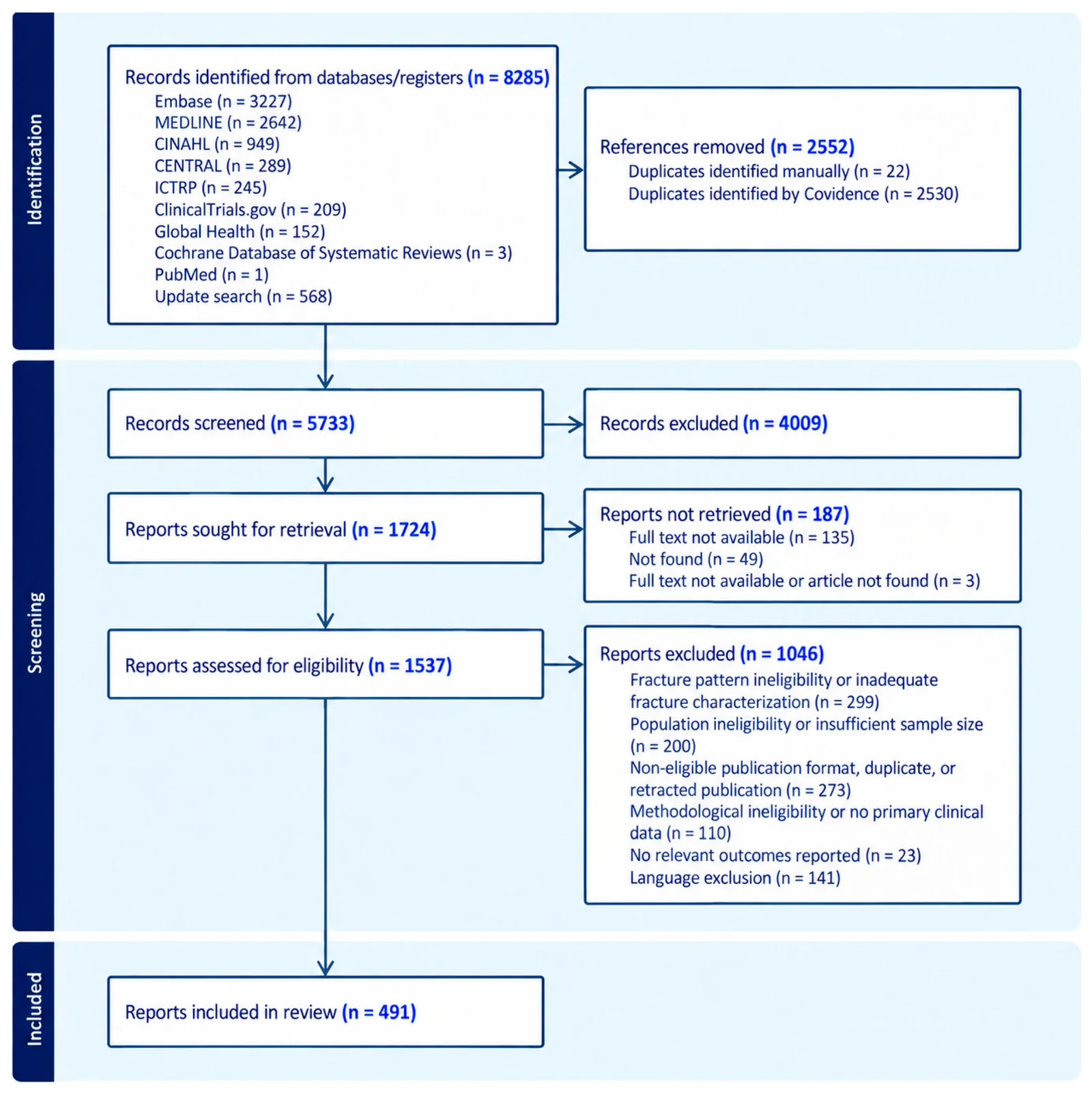
PRISMA 2020 flow diagram for study identification, screening, eligibility assessment, and inclusion. PRISMA = Preferred Reporting Items for Systematic Reviews and Meta-Analyses.

**Figure 2:**
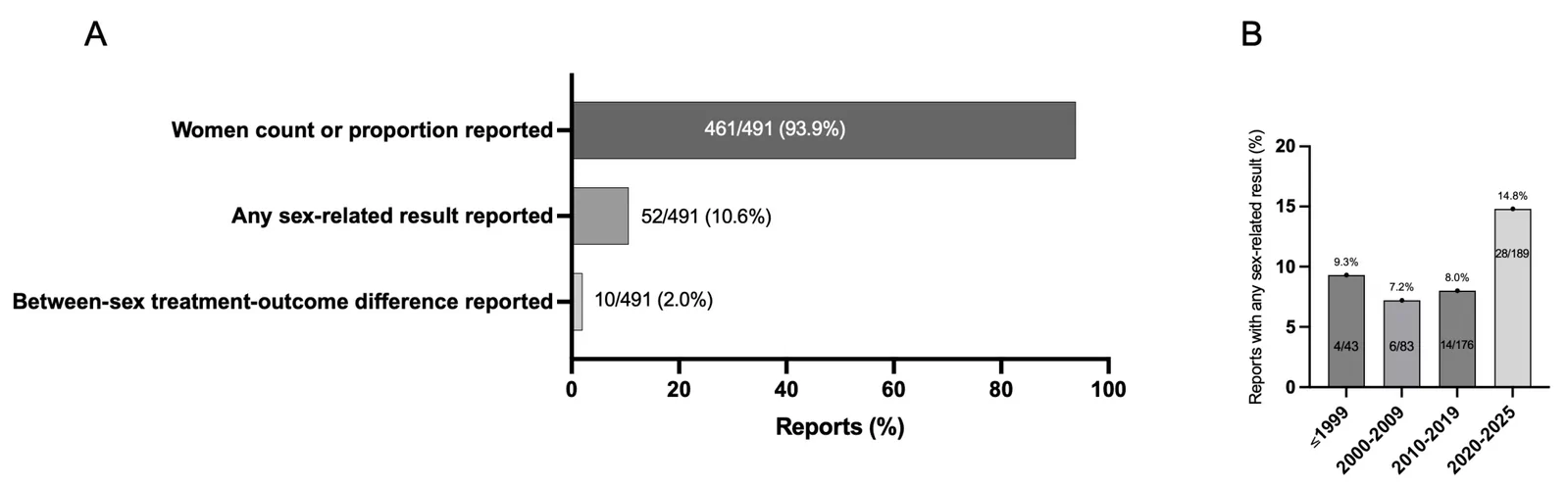
Sex-related reporting in the included thoracolumbar burst fracture literature. (**A**) Reporting cascade showing the proportion of all 491 included reports that documented women count or proportion, reported any sex-related result, and were coded as identifying between-sex outcome differences. (**B**) Proportion of reports with any sex-related result by publication period. The denominator shown inside each column indicates the number of included reports published during that period; together, these period-specific denominators sum to the full set of 491 included reports. Percentages above the columns are calculated within each publication period 4/43 for ≤1999, 6/83 for 2000–2009, 14/176 for 2010–2019, and 28/189 for 2020–2025.

**Table 1:** Characteristics of the included thoracolumbar burst fracture reports.

Domain	Category	*n*	%
Publication period	Range	1979–2025	
Sample size	Median participants (IQR)	50 (30–78)	
	Total included reports	491	
	Total participants	44,147	
Study design	Retrospective	296	60.3
	Prospective nonrandomized	104	21.2
	Randomized controlled trial	50	10.2
	Cohort study	21	4.3
	Other/unclear	20	4.1
Treatment category	Operative techniques	410	83.5
	Operative vs. conservative	42	8.6
	Conservative only	33	6.7
	Other/unclear	6	1.2
Primary region	Asia	239	48.7
	Europe	170	34.6
	North America	54	11.0
	Other/multiregional/unclear	28	5.7

Note: Regional categories were harmonized from the heterogeneous location field in the extraction file and therefore should be interpreted as broad descriptive groupings. The report was the unit of analysis; therefore, the aggregate participant count may include duplicate counting of participants described in companion publications.

**Table 2:** Representation of women and frequency of sex-related reporting in the included reports.

Domain	Indicator	*n*/Summary	%
Representation of women	Women count or proportion reported	461/491	93.9
	Median report-level women proportion (IQR)	36.0 (29.9–43.3)	
	Aggregate proportion of women across reports	12,543/33,906	37.0
	Reports with <40% women	300/461	65.1
	Reports with 40–49.9% women	102/461	22.1
	Reports with ≥50% women	59/461	12.8
Sex-related reporting	Any sex-related result reported	52/491	10.6
	Between-sex outcome difference reported	10/491	2.0
	No between-sex outcome difference reported	181/491	36.9
	Outcome-difference status unknown/not reported	300/491	61.1
Granularity of disaggregated data	Mean age reported separately for women and men	10/491	2.0
	Intervention-outcome mean reported separately for women and men	3/491	0.6

**Table 3:** Regional variation in sex-related reporting and between-sex treatment-outcome differences. The table presents the absolute number and proportion of reports that contain sex-related findings and explicit between-sex outcome differences by geographic region. Percentages were calculated using the number of reports in each regional category as the denominator.

Region	Total Reports	Any Sex-Related Result Reported, *n/N* (%)	Between-Sex Treatment-Outcome Difference Reported, *n/N* (%)
Asia	239	29/239 (12.1%)	3/239 (1.3%)
Europe	170	17/170 (10.0%)	6/170 (3.5%)
North America	54	4/54 (7.4%)	0/54 (0.0%)
Other/multiregional/unclear	28	2/28 (7.1%)	1/28 (3.6%)
Total	491	52/491 (10.6%)	10/491 (2.0%)

**Table 4:** Sex-related treatment-outcome differences reported in the included literature. The table includes all ten reports coded as identifying a between-sex treatment-outcome difference in the final analysis. Findings should be interpreted as hypothesis-generating because study designs, outcomes, and statistical approaches varied substantially.

Study	Outcome/Domain	Main Sex-Related Finding	Interpretation and Limitations
Maior 2018 [7]	HRQOL and disability after surgery	Men had better SF-36 Physical Function, Bodily Pain and Social Function scores and lower ODI disability at 1 year; overall ODI difference favored men (*p* = 0.0028).	Sex/gender was a stated study objective, but comparisons were unadjusted and based on a small surgical cohort.
Dandurand 2025 [6]	ODI recovery; operative vs. nonoperative treatment	Surgically treated women reached ODI MCID faster than men (14 vs. 28 days, *p* = 0.009). Among nonoperatively treated patients, women had a lower probability of ODI improvement (HR 0.55, 95% CI 0.32–0.96).	Prospective multicentre data with multivariable analysis; one of the few studies specifically designed to investigate sex/gender differences.
Lofrese 2022 [17]	Pain, disability and quality of life after SpineJack	Women had higher VAS at follow-up (*p* = 0.02), worse ODI (*p* = 0.04), and lower EQ-5D (*p* = 0.05) than men.	Secondary associations within a retrospective case series; sex was one of several demographic variables examined.
Noriega 2021 [18]	ODI and radiographic correction after PIETI/SpineJack	Men showed greater correction of traumatic regional angulation, whereas improvement in ODI at final follow-up was greater in women.	Small prospective pilot cohort; multiple outcomes and subgroup comparisons.
Ge 2025 [19]	Postoperative kyphosis correction loss	Male sex was independently associated with correction loss (OR 2.201, 95% CI 1.107–4.377; *p* = 0.025).	Adjusted secondary risk-factor analysis; effect derived from the whole mixed fracture cohort rather than an A3/A4-only subgroup.
Jang 2018 [20]	Vertebral body recollapse	Male sex was associated with recollapse on univariable analysis (OR 4.714, *p* = 0.050), but not after adjustment (adjusted OR 2.971, 95% CI 0.565–15.633; *p* = 0.199).	Illustrates that an apparent sex association may disappear after adjustment for other predictors.
Aono 2022 [21]	Fracture reduction angle and reduction rate	Women had greater reduction angle and reduction rate; male sex remained negatively associated with both outcomes in multiple regression (*p* < 0.001 and *p* = 0.003, respectively).	Sex was a secondary predictor in a multivariable radiographic risk-factor analysis.
Caruso 2018 [22]	Segmental and vertebral kyphosis	Sex-related differences in radiographic correction were reported, with greater residual segmental kyphosis in men in the study analyses.	Retrospective analysis; sex was assessed alongside procedure, fracture classification, level and age, without establishing a consistent independent sex effect.
Mohammed 2023 [23]	Cobb-angle correction	Male sex was associated with greater Cobb-angle change: +6.1° (95% CI 0.01–12.1) preoperative-to-postoperative and +8.8° (95% CI 3.8–13.8) preoperative-to-final follow-up.	Secondary regression finding; should not be interpreted as evidence of better overall clinical recovery in men.
Reinhold 2009 [24]	Functional mobility	Women showed better spinal mobility on fingertip-to-floor distance, while most other clinical outcomes were similar between sexes.	Large prospective multicentre follow-up, but the sex-related finding was limited to a selected functional measure rather than a consistent difference across outcomes.

## Data Availability

The de-identified extraction dataset underlying the analyses presented in this review is provided in the Supplementary Materials. The dataset includes the report-level variables used to generate the descriptive analyses, tables, and figures. Additional information can be obtained from the corresponding author upon reasonable request.

## Supplementary Materials

The following supporting information can be downloaded at: https://www.mdpi.com/article/10.3390/jcm15197478/s1, Table S1, full search strategies; Table S2, characteristics of reports identifying between-sex treatment-outcome differences; PRISMA-ScR checklist. Data S1, de-identified extraction dataset used for the final analyses.

## Abbreviations

The following abbreviations are used in this manuscript:

AO	Arbeitsgemeinschaft für Osteosynthesefragen;
CENTRAL	Cochrane Central Register of Controlled Trials;
CINAHL	Cumulative Index to Nursing and Allied Health Literature;
ICTRP	WHO International Clinical Trials Registry Platform;
IQR	interquartile range;
JCM	Journal of Clinical Medicine;
PRISMA-ScR	Preferred Reporting Items for Systematic Reviews and Meta-Analyses Extension for Scoping Reviews;
RCT	randomized controlled trial;
SAGER	Sex and Gender Equity in Research.

## References

[1] Bigdon S.F., Saldarriaga Y., Oswald K.A.C., Müller M., Deml M.C., Benneker L.M., Ecker T.M., Albers C.E. (2022). Epidemiologic Analysis of 8000 Acute Vertebral Fractures: Evolution of Treatment and Complications at 10-Year Follow-Up. J. Orthop. Surg. Res..

[2] Rajasekaran S. (2010). Thoracolumbar Burst Fractures without Neurological Deficit: The Role for Conservative Treatment. Eur. Spine J..

[3] Tanasansomboon T., Kittipibul T., Limthongkul W., Yingsakmongkol W., Kotheeranurak V., Singhatanadgige W. (2022). Thoracolumbar Burst Fracture without Neurological Deficit: Review of Controversies and Current Evidence of Treatment. World Neurosurg..

[4] Salamanna F., Contartese D., Tschon M., Borsari V., Griffoni C., Gasbarrini A., Fini M. (2022). Sex and Gender Determinants Following Spinal Fusion Surgery: A Systematic Review of Clinical Data. Front. Surg..

[5] Heidari S., Babor T.F., De Castro P., Tort S., Curno M. (2016). Sex and Gender Equity in Research: Rationale for the SAGER Guidelines and Recommended Use. Res. Integr. Peer Rev..

[6] Dandurand C., Dvorak M.F., Öner C.F., Hauri D., Schnake K., Vaccaro A.R., Benneker L.M., Schroeder G.D., Rajasekaran S., El-Skarkawi M. (2025). Gender Differences in Outcomes in Patients Treated for Thoracolumbar Burst Fractures Without Neurological Deficits: Prospective International Multicenter Study. Neurosurgery.

[7] Maior T., Ungureanu G., Kakucs C., Berce C., Petrushev B., Florian I.-S. (2018). Influence of Gender on Health-Related Quality of Life and Disability at 1 Year After Surgery for Thoracolumbar Burst Fractures. Glob. Spine J..

[8] Tricco A.C., Lillie E., Zarin W., O’Brien K.K., Colquhoun H., Levac D., Moher D., Peters M.D.J., Horsley T., Weeks L. (2018). PRISMA Extension for Scoping Reviews (PRISMA-ScR): Checklist and Explanation. Ann. Intern. Med..

[9] von Gernler M., Veenstra A.C., Häckel S. (2025). Sexual Differences in the Treatment of Thoracolumbar Burst Fractures. [ClinicalTrials.Gov]. searchRxiv.

[10] von Gernler M., Veenstra A.C., Häckel S. (2025). Sexual Differences in the Treatment of Thoracolumbar Burst Fractures. [Ovid Embase]. searchRxiv.

[11] von Gernler M., Veenstra A.C., Häckel S. (2025). Sexual Differences in the Treatment of Thoracolumbar Burst Fractures. [Cochrane Library]. searchRxiv.

[12] von Gernler M., Veenstra A.C., Häckel S. (2025). Sexual Differences in the Treatment of Thoracolumbar Burst Fractures. [Ovid MEDLINE]. searchRxiv.

[13] von Gernler M., Veenstra A.C., Häckel S. (2025). Sexual Differences in the Treatment of Thoracolumbar Burst Fractures. [ICTRP]. searchRxiv.

[14] von Gernler M., Veenstra A.C., Häckel S. (2025). Sexual Differences in the Treatment of Thoracolumbar Burst Fractures. [CINAHL]. searchRxiv.

[15] von Gernler M., Veenstra A.C., Häckel S. (2025). Sexual Differences in the Treatment of Thoracolumbar Burst Fractures. [Ovid Global Health]. searchRxiv.

[16] Borissov N., Haas Q., Minder B., Kopp-Heim D., von Gernler M., Janka H., Teodoro D., Amini P. (2022). Reducing Systematic Review Burden Using Deduklick: A Novel, Automated, Reliable, and Explainable Deduplication Algorithm to Foster Medical Research. Syst. Rev..

[17] Lofrese G., Ricciardi L., De Bonis P., Cultrera F., Cappuccio M., Scerrati A., Martucci A., Musio A., Tosatto L., De Iure F. (2022). Use of the SpineJack Direct Reduction for Treating Type A2, A3 and A4 Fractures of the Thoracolumbar Spine: A Retrospective Case Series. J. Neurointerventional Surg..

[18] Noriega D.C., Crespo-Sanjuan J., Olan W.J., Hernandez-Ramajo R., Bell D.P., Sanz J.J.C., Hernandez G.d.J.L., Sanchez-Lite I., Ardura F. (2021). Treatment of Thoracolumbar Type A3 Fractures Using a Percutaneous Intravertebral Expandable Titanium Implant: Long-Term Follow-up Results of a Pilot Single Center Study. Pain Physician.

[19] Ge T., Li W., Wu J., Wang Q., Li C., Wang S., Xiong W., Fan J. (2025). Impact of Traumatic Intervertebral Disc Injury on Loss of Correction Following Pedicle Screw Fixation for Thoracolumbar Fractures. BMC Musculoskelet. Disord..

[20] Jang H.-D., Bang C., Lee J.C., Soh J.-W., Choi S.-W., Cho H.-K., Shin B.-J. (2018). Risk Factor Analysis for Predicting Vertebral Body Re-Collapse after Posterior Instrumented Fusion in Thoracolumbar Burst Fracture. Spine J..

[21] Aono H., Takenaka S., Okuda A., Kikuchi T., Takeshita H., Nagata K., Ito Y. (2022). Risk Factors for Insufficient Reduction after Short-Segment Posterior Fixation for Thoracolumbar Burst Fractures: Does the Interval from Injury Onset to Surgery Affect Reduction of Fractured Vertebrae?. J. Orthop. Surg. Res..

[22] Caruso G., Lombardi E., Andreotti M., Lorusso V., Gildone A., Padovani S., Massari L. (2018). Minimally Invasive Fixation Techniques for Thoracolumbar Fractures: Comparison between Percutaneous Pedicle Screw with Intermediate Screw (PPSIS) and Percutaneous Pedicle Screw with Kyphoplasty (PPSK). Eur. J. Orthop. Surg. Traumatol..

[23] Mohammed R., Carrasco R., Verma R., Siddique I., Mohammad S., Elmalky M. (2023). Does Instrumentation of the Fractured Level in Thoracolumbar Fixation Affect the Functional and Radiological Outcome?. Glob. Spine J..

[24] Reinhold M., Knop C., Beisse R., Audigé L., Kandziora F., Pizanis A., Pranzl R., Gercek E., Schultheiss M., Weckbach A. (2009). [Operative Treatment of Traumatic Fractures of the Thoracic and Lumbar Spinal Column: Part III: Follow up Data]. Der Unfallchirurg.

[25] Compston J.E., McClung M.R., Leslie W.D. (2019). Osteoporosis. Lancet.

[26] Schömig F., Becker L., Schönnagel L., Völker A., Disch A.C., Schnake K.J., Pumberger M. (2023). Avoiding Spinal Implant Failures in Osteoporotic Patients: A Narrative Review. Glob. Spine J..

[27] Clayton J.A., Tannenbaum C. (2016). Reporting Sex, Gender, or Both in Clinical Research?. JAMA.

