# Supplementary material for: Sex-Based Representation and Outcome Reporting in Thoracolumbar Burst Fracture Research: A PRISMA-ScR Scoping Review
Source: J Clin Med. 2026 Sep 25;15(19):7478. doi: 10.3390/jcm15197478 (PMC13665723; doi:10.3390/jcm15197478)
Supplement: Supplementary file 1 [file jcm-15-07478-s001.zip › Table S1.pdf]

Embase <1974 to 2024 September 30>

1 exp thoracolumbar fracture/ 1048

2 thoracolumbar spine/ or (exp thoracic spine/ and exp lumbar spine/) 14401

3 spine fracture/ or exp burst fracture/ or compression fracture/ or exp lumbar spine fracture/ or exp thoracic spine fracture/ or vertebral body fracture/ or vertebral pedicle fracture/ 34399

4 2 and 3 3338

5 ((thor*lumba? or thor*-lumba?) adj3 (injur* or fractur* or trauma* or compressio* or burst)).ti,ab,kf. 5003

6 (trauma* adj3 fractur* adj3 spine).ti,ab. 299

7 or/1,4-6 [population spinal/thoracolumbar fractures] 6992

8 conservative treatment/ or bed rest/ or spinal cord decompression/ or spine stabilization/ or exp spine fusion/ or exp traction therapy/ or watchful waiting/ 194494

9 minimally invasive procedure/ or minimally invasive surgery/ or minimally invasive spine surgery/ 82943

10 ((surger* or surgeo* or surgic* or nonsurg* or operative* or nonoperative* or conservative*) adj4 (treatment* or therap* or technique*)).ti,ab. 696966

11 (minimal* adj4 surg#*).ti,ab. 53798

12 (spondylodes* or spondylosyndes* or ((segment or spin* or fracture*) adj4 (fixation* or fusion*))).ti,ab. 47769

13 ((short or long or posterior or anterior) adj3 fusion).ti,ab. 17860

14 exp vertebral screw/ or exp spine fixation device/ or percutaneous vertebroplasty/ or kyphoplasty/ or kyphoplasty system/ 26710

15 (((spinal or dorsal or posterior) adj2 instrumentation) or pedicle screw? or vertebroplasty or kyphoplasty or OsseoFix or Spinejack or orthos?s).ti,ab. 33660

16 ("Altius M-INI Spinal System" or "Altius OCT" or "Altius OCT Spinal System" or "Altius Oct Spine System" or "ANAXTM5.5 Spinal System" or "ANAXTMOCT Spinal System" or "ATLANTIS VISION" or "AXIS Fixation System" or "bone screw spinal fixation system" or "bone-screw internal spinal fixation system" or "Caspian Occipital Anchor" or "CD Horizon Essence" or "Centerline" or "Chesapeake" or "ConneX Cross Connector" or "ConneX Rod Connector" or "Conquest Spinal System" or "Cypress" or "Danek Unit Rod" or "Daytona" or "Denali MI" or "Denali Mini" or "Dymaxeon" or "DYNA-LOK Spinal System" or "Dynabolt" or "Easyspine" or "EQUATION Fixation System" or "ES2" or

"Excella MIS" or "FacetLINK" or "Firebird Deformity Correction System" or "Firebird Nxg" or "Firebird SFS" or "Firebird Spinal Fixation System" or "FixxSure" or "flamenco spinal fixation system" or "GDLH Posterior Spinal System" or "Gibralt Spine System" or "HydraLok" or "Instinct" or "Isobar" or "JANUS" or "Janus" or "Liberty" or "LOSPA IS Spinal Fixation System" or "LumFix Spinal Fixation System" or "Macs Ii" or "Mantis" or "Medacta Unconstrained Screw Technology" or "Mega Plus Spine System" or "Mesa" or "Mesa 2 Deformity" or "Mesa Deformity" or "Mesa Degenerative" or "Mesa Mini" or "Mesa Small Stature" or "ModuLIF" or "Monopoly Vc" or "Natural Bridge Low Profile" or "Natural Bridge Mini" or "Nautilus" or "NFlex" or "non-sterile bone-screw internal spinal fixation system" or "Oak" or "Odalys" or "Percudyn System" or "Percusys" or "Phoenix" or "Phoenix CDX" or "Proliant" or "Quantum Flex" or "Reflect" or "Rexious" or "Salvo Spine System" or "Savannah-T Dual Fix" or "SCS CLARIS Spinal Screws" or "Securis" or "Silhouette Spinal System" or "SiLo" or "spinal pedicle fixation device" or "spine pedicle fixation device" or "Spinelink - Ii Spinal Fixation System" or "sterile bone-screw internal spinal fixation system" or "Tangent Plus" or "Threaded CR-Ti" or "TiTLE" or "TiTLE NorthStar" or "Trinica" or "Trio Plus" or "TSRH Spinal System" or "TSRH-3D" or "Universal Spine System" or "USS" or "USS II" or "Vantage Anterior Fixation System" or "Venture Anterior Cervical Plate System" or "Vertex Reconstruction System" or "Vertu Ti-Bond" or "VIPER 2" or "Viper Prime" or "ZPLATE II Anterior Fixation System" or "pedicle screw fixation device" or "Everest MI" or "OsseoScrew").ti,ab,dm,dv,kf,my. 462442

17 ("ARCH" or "Bridgepoint" or "CD HORIZON BalanC" or "CD Horizon Legacy" or "CD Horizon M8" or "CD Horizon Sextant" or "CD Horizon Solera" or "CD Horizon Spinal System" or "CDH-M8" or "Centerpiece" or "Centurion" or "Clutch" or "Escalate" or "Expedium" or "Expedium Verse" or "external spinal fixation system" or "ISOLA" or "ISOLA" or "Karma" or "Lotus" or "Matrix" or "Mercury" or "Mercury" or "Moss Miami" or "Mountaineer OCT" or "Nile" or "Overwatch" or "spinal fixation device" or "spinal fixation systems" or "spinal fixator" or "spinal stabilisation device" or "spinal stabilization device" or "spine stabilisation device" or "spine stabilization device" or "SpineJack" or "Symphony" or "Vertex Select" or "VIPER" or "spine fixation device").ti,ab,dm,dv,kf,my. 766845

18 ("AVAflex" or "balloon kyphoplasty kit" or "CementoFixx-M" or "InflateFX Gen II" or "InterV Flex Catheter" or "Kyphon" or "KYPHON Express II" or "kyphoplasty balloon" or "kyphoplasty balloon catheter" or "kyphoplasty device" or "KyphPak First Fracture" or "Osseoflex SB" or "Osseoflex SN" or "StabiliT MX" or "StabiliT VP" or "Synflate" or "kyphoplasty system").ti,ab,dm,dv,kf,my. 341

19 or/8-18 [Intervention: surgical/nonsurgical treatment] 2154830

20 quality of life/ or quality adjusted life year/ or exp quality of life assessment/ 778514

21 (health-related quality of life or HRQOL or life quality or quality of life or QoL or Quality-adjusted life years or QALYs).ti,ab. 673634

22 (short form-36 or SF-36 or short form-12 or SF-12 or EuroQol-5D or EQ-5D or EuroQol-5D-5L or EQ-5D-5L).ti,ab. 85808

23 length of stay/ 299353

24 (hospital stay or length of stay).ti,ab. 323663

25 operation duration/ 161185

26 ("operati* time" or "surgical time" or "length of surgery" or "operati* duration" or "surgery duration" or "duration of surgery" or "time in surgery").ti,ab. 149074

27 operative blood loss/ 42889

28 (surgical blood loss or surgical h?emorrhag* or operative blood loss or operative h?emorrhag*).ti,ab. 6520

29 exp backache/ 146016

30 (back ache or back pain or backache or vertebrogenic pain syndrome or dorsalgia).ti,ab. 92083

31 exp treatment outcome/ 2728256

32 (treatment outcome or clinical outcome or clinical effectiveness or clinical efficacy or patient-relevant outcome or rehabilitation outcome or treatment effectiveness or treatment efficacy or therap* outcome).ti,ab. 349377

33 (patient reported outcome* or patientreported outcome* or patient reported treatment outcome* or self-reported outcome* or self-reported patient outcome* or self-reported treatment outcome* or selfreported outcome* or patient outcome assessment or patient-centered outcomes research or PROMs or Patient-reported spine trauma outcome measure or PROST or Patient-Reported Outcomes Measurement Information System or PROMIS or overall satisfaction with the treatment).ti,ab. 73732

34 exp postoperative pain/ 96213

35 ("postoperative pain*" or "pain after operation" or "pain after surgery" or "post-operation pain*" or "post-surgery pain*" or "post-surgical pain*" or "postoperation pain*" or "postoperative pains*" or "postsurgery pain*" or "postsurgical pain*" or "surgery pain*" or "surgery-associated pain*" or "surgery-derived pain*" or "surgery-induced pain*" or "surgery-related pain*" or "surgical pain*").ti,ab. 54262

36 incremental cost effectiveness ratio.dq. 391

37 (incremental cost-effectiveness ratio or ICER or indirect cost questionnaire).ti,ab. 19761

38 visual analog scale/ 138504

39 pain measurement/ or pain assessment/ or visual analog scale/ or numeric rating scale/ or dallas pain questionnaire.dq. 250813

40 (visual analog scale* or numeric rating scale or dallas pain questionnaire).ti,ab. 64355

41 disability assessment/ or neck disability index/ or oswestry disability index/ or roland morris disability questionnaire/ or quebec back pain disability scale.dq. 20550

42 ("Oswestry Disability Index" or "Oswestry Disability Questionnaire" or "Oswestry Index" or "Oswestry low back pain disability index" or "Oswestry low back pain disability questionnaire" or "Oswestry Questionnaire" or "Oswestry scale" or "Oswestry score" or "Oswestry scores" or "Roland Morris Disability" or "neck pain disability index" or "Neck Disability Index" or "quebec back pain disability" or "functional disabilit*").ti,ab. 32391

43 American Spinal Injury Association impairment scale/ or Frankel grade/ 3783

44 ("American Spinal Injury Association grade" or "American Spinal Injury Association motor score" or "American Spinal Injury Association score" or "ASIA impairment scale" or "ASIA motor score" or "ASIA scale" or "ASIA score" or "American Spinal Injury Association impairment scale" or "Frankel classification" or "Frankel score" or "Frankel grade").ti,ab. 3771

45 (Core Outcome Measures Index or COMI or short musculoskeletal function assessment or SMFA or low back outcome score or low back outcome scale).ti,ab,dq. 1184

46 exp kyphosis/ or lordosis/ or exp Cobb angle/ 35384

47 (((kyphos#s or hyperkyphos#s or (Gardner or Cobb or kyphotic)) adj2 angle) or round back or hunchback or lordos#s).ti,ab. 21483

48 vertebral height/ or adjacent segment disease/ or fracture nonunion/ 20333

49 (Sagittal balance or Coronal balance or Vertebral body height or adjacent segment degeneration or Canal compromise or Disc space height or Implant loosening or non-union or malunion or fracture compression or vertebral body displacement or Mc Cormack or Posterior ligamentous complex integrity or vertebral body height ratio or bone fragments in the spinal canal or degeneration of the intervertebral disc or Pfirrmann or Spine Adverse Events Severity System).ti,ab,dq. 20547

50 reoperation/ 112027

51 (reoperation or revision surgery or revision surgeries or surgical revision or joint revision or repeat surgery or re-operation or re-operative intervention or re-operative operation or re-operative repair or re-operative surgery or re-surgery or redo operation or redo surgery or redo surgical intervention or redo surgical procedure or redo surgical repair or redo surgical replacement or reoperative surgery).ti,ab. 94034

52 surgical infection/ 68184

53 ((operative or postoperative or surgical or surgery) adj3 infect*).ti,ab. 70584

54 vein thrombosis/ or deep vein thrombosis/ 122783

55 (deep vein thrombosis or deep vein thromboses).ti,ab. 34533

56 return to work/ or medical leave/ 18954

57 (return to work or back to work or sick leave or sick days or sick day or illness day or illness days or medical leave or disability leave or disability leaves).ti,ab. 25411

58 or/20-57 [Outcomes] 4581339

59 and/7,19,58 3237

60 ("12091034" or "12486347" or "12800001" or "15087804" or "15682000" or "15699801" or "16622372" or "16703901" or "16850952" or "17054237" or "17139218" or "17252216" or "17566793" or "18193300" or "18312076" or "19165509" or "19320579" or "20417512" or "22395303" or "22844053" or "22890568" or "23584396" or "23732185" or "23740669" or "24018981" or "24170266" or "24321130" or "24358996" or "24509773" or "25108675" or "25263584" or "25688302" or "25757843" or "26051881" or "26097844" or "26907876" or "26933621" or "26983411" or "27496720" or "27554351" or "27751172" or "27757682" or "28399557" or "28571219" or "28807432" or "29071409" or "29151568" or "29356907" or "29678706" or "30167741" or "31048604" or "31418065" or "31431406" or "31695276" or "32036427" or "32208941" or "32347391" or "32802519" or "33066988" or "34228696" or "35144416" or "38493890" or "21603733" or "26516731").pm. 58

61 and/59-60 52

62 (exp animal/ or exp animal experiment/ or exp animal model/ or exp plant/ or exp fungus/) not exp human/ 6674067

63 59 not 62 3209

https://ovidsp.ovid.com/ovidweb.cgi?T=JS&NEWS=N&PAGE=main&SHAREDSEARCHID=8YHuRwc3MFsnsVYlVqHPvsAt6kYzt28zkTbtChoBMnycY9vlnnUejgs0EnYyEkJC

Ovid MEDLINE(R) ALL <1946 to September 23, 2024>

1 (*lumbar vertebrae/ or *thoracic vertebrae/) and (Spinal Fractures/ or Fractures, Comminuted/ or Fractures, Compression/) 4073

2 ((thor*lumba? or thor*-lumba?) adj3 (injur* or fractur* or trauma* or compressio* or burst)).ti,ab,kf. 3734

3 (trauma* adj3 fractur* adj3 spine).ti,ab. 215

4 or/1-3 [Population: spinal/thoracolumbar fractures] 6521

5 conservative treatment/ or minimally invasive surgical procedures/ or spinal fusion/ or fracture fixation/ or exp fracture fixation, internal/ 136755

6 ((surger* or surgeo* or surgic* or nonsurg* or operative* or nonoperative* or conservative*) adj4 (treatment* or therap* or technique*)).ti,ab. 524517

7 (minimal* adj4 surg#*).ti,ab. 35543

8 (spondylodes* or spondylosyndes* or ((segment or spin* or fracture*) adj4 (fixation* or fusion*))).ti,ab. 39485

9 ((short or long or posterior or anterior) adj3 fusion).ti,ab. 14221

10 Pedicle Screws/ or Vertebroplasty/ or Kyphoplasty/ 6837

11 (((spinal or dorsal or posterior) adj2 instrumentation) or pedicle screw? or vertebroplasty or kyphoplasty or OsseoFix or Spinejack or orthos?s).ti,ab. 25230

12 or/5-11 [Intervention: surgical/nonsurgical treatment] 680002

13 Quality of Life/ 293829

14 (health-related quality of life or HRQOL or life quality or quality of life or QoL or Quality-adjusted life years or QALYs).ti,ab. 425019

15 (short form-36 or SF-36 or short form-12 or SF-12 or EuroQol-5D or EQ-5D or EuroQol-5D-5L or EQ-5D-5L).ti,ab. 52250

16 Length of Stay/ 106758

17 (hospital stay or length of stay).ti,ab. 182925

18 Operative Time/ 19050

19 ("operati* time" or "surgical time" or "length of surgery" or "operati* duration" or "surgery duration" or "duration of surgery" or "time in surgery").ti,ab. 91692

20 Blood Loss, Surgical/ 21242

21 (surgical blood loss or surgical h?emorrhag* or operative blood loss or operative h?emorrhag*).ti,ab. 4180

22 Back Pain/ 19351

23 (back ache or back pain or backache or vertebrogenic pain syndrome or dorsalgia).ti,ab. 64966

24 Treatment Outcome/ 1210457

25 (treatment outcome or clinical outcome or clinical effectiveness or clinical efficacy or patient-relevant outcome or rehabilitation outcome or treatment effectiveness or treatment efficacy or therap* outcome).ti,ab. 224932

26 Postoperative Complications/ 413822

27 ("postoperative complication*" or "complication after operation" or "complication after surgery" or "post-operation complication*" or "post-surgery complication*" or "post-surgical complication*" or "postoperation complication*" or "postoperative complications*" or "postsurgery complication*" or "postsurgical complication*" or "surgery complication*" or "surgery-associated complication*" or "surgery-derived complication*" or "surgery-induced complication*" or "surgery-related complication*" or "surgical complication*").ti,ab. 114834

28 Pain, Postoperative/ 51245

29 ("postoperative pain*" or "pain after operation" or "pain after surgery" or "post-operation pain*" or "post-surgery pain*" or "post-surgical pain*" or "postoperation pain*" or "postoperative pains*" or "postsurgery pain*" or "postsurgical pain*" or "surgery pain*" or "surgery-associated pain*" or "surgery-derived pain*" or "surgery-induced pain*" or "surgery-related pain*" or "surgical pain*").ti,ab. 39105

30 Patient Outcome Assessment/ or Patient Reported Outcome Measures/ 22413

31 (patient reported outcome* or patientreported outcome* or patient reported treatment outcome* or self-reported outcome* or self-reported patient outcome* or self-reported treatment outcome* or selfreported outcome* or patient outcome assessment or patient-centered outcomes research or PROMs or Patient-reported spine trauma outcome measure or PROST or Patient-Reported Outcomes Measurement Information System or PROMIS or overall satisfaction with the treatment).ti,ab. 43106

32 (incremental cost-effectiveness ratio or ICER or indirect cost questionnaire).ti,ab. 10879

33 visual analog scale/ 4171

34 (visual analog scale* or numeric rating scale or dallas pain questionnaire).ti,ab. 48402

35 (oswestry disability index or quebec back pain disability scale or roland-morris disability questionnaire or neck disability index or functional disabilit*).ti,ab. 22275

36 (american spinal injury association impairment scale or frankel classification).ti,ab. 957

37 (Core Outcome Measures Index for the Spine or COMI or Short Musculoskeletal Function Assessment or SMFA or Low Back Outcome Scale).ti,ab. 687

38 kyphosis/ or lordosis/ 10905

39 (((kyphos#s or hyperkyphos#s or (Gardner or Cobb or kyphotic)) adj2 angle) or round back or hunchback or lordos#s).ti,ab. 16261

40 (Sagittal balance or Coronal balance or Vertebral body height or adjacent segment degeneration or Canal compromise or Disc space height or Implant loosening or non-union or malunion or fracture compression or vertebral body displacement or Mc Cormack or Posterior ligamentous complex integrity or vertebral body height ratio or bone fragments in the spinal canal or degeneration of the intervertebral disc or Pfirrmann or Spine Adverse Events Severity System).ti,ab. 16380

41 reoperation/ 97816

42 (reoperation or revision surgery or revision surgeries or surgical revision or joint revision or repeat surgery).ti,ab. 60581

43 surgical wound infection/ 41855

44 (surgical wound infection or rates of infection).ti,ab. 4001

45 Venous Thrombosis/ 30698

46 (deep vein thrombosis or deep vein thromboses).ti,ab. 21557

47 Return to Work/ or Sick Leave/ 10163

48 (return to work or back to work or sick leave or sick days or sick day or illness day or illness days or disability leave or disability leaves).ti,ab. 18862

49 Survival Rate/ or Morbidity/ 225125

50 ("mean survival time" or "mean survival times" or "survival rate" or "survival rates" or "morbidity").ti,ab. 669744

51 or/13-50 [Outcomes] 3220579

52 and/4,12,51 2601

53 (exp animals/ or exp animal experimentation/ or exp models, animal/ or exp plants/ or exp fungi/) not humans/ 5709084

54 52 not 53 2589

55 ("12091034" or "12486347" or "12800001" or "15087804" or "15682000" or "15699801" or "16622372" or "16703901" or "16850952" or "17054237" or "17139218" or "17252216" or "17566793" or "18193300" or "18312076" or "19165509" or "19320579" or "20417512" or "22395303" or "22844053" or "22890568" or "23584396" or "23732185" or "23740669" or "24018981" or "24170266" or "24321130" or "24358996" or "24509773" or "25108675" or "25263584" or "25688302" or "25757843" or "26051881" or "26097844" or "26907876" or "26933621" or "26983411" or "27496720" or "27554351" or "27751172" or "27757682" or "28399557" or "28571219" or "28807432" or "29071409" or "29151568" or "29356907" or "29678706" or "30167741" or "31048604" or "31418065" or "31431406" or "31695276" or "32036427" or "32208941" or "32347391" or "32802519" or "33066988" or "34228696" or "35144416" or "38493890" or "21603733" or "26516731").ui. 64

56 54 and 55 60
