# Supplementary material for: Sex-Based Representation and Outcome Reporting in Thoracolumbar Burst Fracture Research: A PRISMA-ScR Scoping Review
Source: J Clin Med. 2026 Sep 25;15(19):7478. doi: 10.3390/jcm15197478 (PMC13665723; doi:10.3390/jcm15197478)
Supplement: Supplementary file 1 [file jcm-15-07478-s001.zip › Table S2.pdf]

Table S2. Characteristics of reports identifying between-sex treatment-outcome differences

Study	Design; women/men	Fracture population / treatment	Sex-related outcome and reported effect	Analysis context
**Maior 2018 [7]**	Case series; 17/27	Single-level A3, T12–L2; posterior fixation	Men had better SF-36 Physical Function (p=0.016), Bodily Pain (p=0.003), Social Function (p=0.004), and lower ODI (p=0.003)	Sex/gender was a primary study objective; unadjusted comparisons.
**Dandurand 2025 [6]**	Prospective international multicentre; 68/130	Neurologically intact A3/A4 burst fractures; operative vs nonoperative	Surgical: ODI MCID reached earlier in women (14 vs 28 days, p=0.009). Nonoperative: lower probability of ODI improvement in women (HR 0.55, 95% CI 0.32–0.96)	Dedicated sex/gender study; multivariable analysis.
**Lofrese 2022 [17]**	Retrospective case series; 24/33	A2/A3/A4 T10–L2; SpineJack; A3/A4 50/57	Women had higher VAS (p=0.02), worse ODI (p=0.04) and lower EQ-5D (p=0.05) at follow-up	Secondary sex analysis; not a dedicated sex-specific study.
**Noriega 2021 [18]**	Prospective pilot cohort; 29/15	A3; PIETI/SpineJack	Men showed greater correction of traumatic regional angulation; ODI improvement at final follow-up was greater in women	Secondary subgroup analysis; exact adjusted sex effect/CI not available in the extraction.
**Ge 2025 [19]**	Retrospective cohort; 86/108	Mixed A2/A3/A4/B2/C; A3/A4 118/194; posterior fixation	Male sex independently associated with correction loss (OR 2.201, 95% CI 1.107–4.377; p=0.025)	Secondary multivariable risk-factor analysis; whole mixed cohort.
**Jang 2018 [20]**	Retrospective comparative study; 104/104	T11–L2 traumatic burst fractures; posterior instrumented fusion	Male sex: univariable OR 4.714, p=0.050; adjusted OR 2.971, 95% CI 0.565–15.633; p=0.199 for recollapse	Secondary predictor; sex was not independently associated after adjustment.
**Aono 2022 [21]**	Retrospective multicentre; 113/140	A3/A4/B1/B2; A3/A4 164/253; short-segment fixation	Women had greater reduction angle/rate; male coefficients −1.926 (p<0.001) and −9.424 (p=0.003)	Secondary multivariable analysis; models included age, LSS, preoperative VBA and other factors.
**Caruso 2018 [22]**	Retrospective comparative study; 17/29	Thoracolumbar fractures; PPSIS vs PPSK	Greater residual segmental kyphosis was reported in men	Secondary analysis; exact sex-specific effect estimate/CI not available in the extraction.
**Mohammed 2023 [23]**	Retrospective comparative study; 22/31	Single-level T11–L1; 34 type A/19 type B; fixation with vs without fracture-level screws	Male coefficient for Cobb-angle change +6.1° (95% CI 0.01–12.1) postoperatively and +8.8° (95% CI 3.8–13.8) at final follow-up; p≤0.05	Secondary regression finding; A3/A4 subtypes were not separately reported.
**Reinhold 2009 [24]**	Prospective multicentre follow-up; 236/402	Mixed thoracic/lumbar injuries; operative, conservative and vertebral augmentation cohorts	Fingertip-to-floor distance 9.4 cm in women vs 13.3 cm in men; p<0.05	Secondary functional analysis; sex was one of several factors assessed.

Abbreviations: CI, confidence interval; HR, hazard ratio; MCID, minimal clinically important difference; ODI, Oswestry Disability Index; OR, odds ratio; VAS, visual analogue scale; VBA, vertebral body angle. "Primary" or "dedicated" indicates that sex/gender differences formed an explicit study objective; otherwise, sex was assessed as a secondary or exploratory variable. NR indicates information not reported or not available in the extracted data.
